# Corn Bioethanol Side Streams: A Potential Sustainable Source of Fat-Soluble Bioactive Molecules for High-Value Applications

**DOI:** 10.3390/foods9121788

**Published:** 2020-12-02

**Authors:** Gabriella Di Lena, Jose Sanchez del Pulgar, Ginevra Lombardi Boccia, Irene Casini, Stefano Ferrari Nicoli

**Affiliations:** CREA Research Centre for Food and Nutrition, Via Ardeatina 546, 00178 Rome, Italy; jose.sanchezdelpulgar@crea.gov.it (J.S.d.P.); g.lombardiboccia@crea.gov.it (G.L.B.); irene.casini@crea.gov.it (I.C.); stefano.nicoli@crea.gov.it (S.F.N.)

**Keywords:** bioethanol co-products, post-fermentation corn oil, distiller’s corn oil, thin stillage, by-products, valorization, bioactive molecules, phytosterols, squalene, tocopherols, tocotrienols, tocols, carotenoids

## Abstract

This paper reports data from a characterization study conducted on the unsaponifiable lipid fraction of dry-grind corn bioethanol side streams. Phytosterols, squalene, tocopherols, tocotrienols, and carotenoids were quantified by High Performance Liquid Chromatography with Diode-Array Detector (HPLC-DAD) and Liquid Chromatography-tandem Mass Spectrometry (LC-MS/MS) in different lots of post-fermentation corn oil and thin stillage collected from a bioethanol plant over a time-span of one year. Fat-soluble bioactives were present at high levels in corn oil, with a prevalence of plant sterols over tocols and squalene. Beta-sitosterol and sitostanol accounted altogether for more than 60% of total sterols. The carotenoid profile was that typical of corn, with lutein and zeaxanthin as the prevalent molecules. The unsaponifiable lipid fraction profile of thin stillage was qualitatively similar to that of post-fermentation corn oil but, in quantitative terms, the amounts of valuable biomolecules were much lower because of the very high dilution of this side stream. Results indicate that post-fermentation corn oil is a promising and sustainable source of health-promoting bioactive molecules. The concomitant presence of a variegate complex of bioactive molecules with high antioxidant potentialities and their potential multifaceted market applications as functional ingredients for food, nutraceutical, and cosmeceutical formulations, make the perspective of their recovery a promising strategy to create new bio-based value chains and maximize the sustainability of corn dry-grind bioethanol biorefineries.

## 1. Introduction

Ensuring the access to affordable, reliable, and sustainable energy is one of the Sustainable Development Goals of the 2030 Agenda for Sustainable Development adopted by the United Nations General Assembly in 2015. Biofuels are sustainable and renewable alternatives to fossil fuels, with the advantage of lower carbon and greenhouse gas (GHG) emissions. Global fuel ethanol production reached 115 billion L in 2019, with United States and Brazil, accounting altogether for over 80% of the world production, as the top producers [1]. In Europe, bioethanol, after biodiesel, is the second contributor of renewable energy sources to the transport sector, with a production capacity that in 2019 reached 9.9 billion L, corresponding to over 72% GHG savings [2].

To increase the competitiveness and sustainability of biofuels with respect to fossil fuels, a significant reduction of their production costs is necessary. A promising approach is the valorization of co-products and side streams arising from biofuels production and the maximization of the biomass-to-products value chains by creating biofuel-driven biorefineries.

Ethanol biorefineries are essential drivers of the energy transition, as they convert biomass into low-carbon fuels and also into a range of other valuable low-carbon co-products that may be conveyed to new value chains. Bioethanol production is mainly provided by first-generation biorefineries, with corn as the major feedstock [3]. The recovery of valuable compounds from corn bio-ethanol co-products and their application as ingredients for high-value market products, may open opportunities for the creation of new bio-based value chains. This approach is in line with the principles of circular economy, aiming at ensuring high quality, functional, and safe products to all, while reducing carbon and environmental footprints [4,5]. Although the present work has been conducted in the framework of the European Union’s Horizon 2020-BBI-JU Program (Project EXConsEED, GA n. 792054), the approach proposed has a global interest, with a high potential impact for the big ethanol producers outside Europe, especially United States, a key player in corn bioethanol production.

In the food and feed industry sectors the development of bio-based products is driven by increasing business and consumers’ demand for healthy, green, and sustainable products. The global demand of proteins and health-promoting bioactive substances for the food, feed, and nutraceutical market is steadily increasing. With the actual prospects of a growing world population and the increasing needs of proteins and highly nutrient food within the next decades, largely surpassing the current production capabilities and natural resources, the individuation of new natural and sustainable sources of nutrients and functional ingredients for the food and feed sectors represents a challenge.

Agrifood residues and wastes are potential sustainable sources of proteins and bioactive molecules. Research conducted in the past decade has shown that plant-derived wastes and by-products may be exploited for the development of functional food products [6,7,8,9].

First-generation biofuel biorefineries may be a starting point for new bio-based value chains. Their by-products and side streams still retain a series of valuable bioactive compounds that, if properly recovered and valorized, may open new perspectives for integrated biorefinery systems with the concomitant result of increasing the competitiveness and maximizing the efficiency of the biofuel production process.

Corn, the most common feedstock for bioethanol production in Europe, is inherently rich of bioactive phytochemicals, i.e., plant sterols, tocopherols, tocotrienols, phenolic compounds, and carotenoids [10]. These bioactives withstand the industrial processes of fuel ethanol production and may be found in the side streams together with yeast residues and metabolites resulting from the fermentation process. The dry-grind corn bioethanol production process gives two side streams, post-fermentation corn oil and thin stillage, currently not fully valorized. Post-fermentation corn oil, obtained by centrifugation of corn syrup, may be utilized for bio-diesel production. However, its full potentialities are actually unexploited since molecules other than fatty acids, reported to hinder the efficiency of biodiesel production, remain unused, while could first be recovered and conveyed to high-end applications.

Thin stillage, obtained after centrifugation of thick/whole stillage, is a highly diluted stream containing suspended particles and dissolved nutrients that originate from spent corn grains and yeast cells in bioethanol biorefineries. Currently, its main use in the feed sector mixed to distiller’s dried grains with solubles (DDGS) requires high energy-demanding evaporation steps that reduce the economic sustainability of the whole biotech process.

With a circular economy approach the bio-molecules present in corn ethanol side streams, phytosterols, phenolics, tocols, squalene, and carotenoids, retaining health protective properties (i.e., antioxidant, anti-inflammatory and anti-aging) could be recovered and valorized by their reintroduction in productive processes as functional ingredients of high-value products (food, nutraceutics, cosmetics). The first step to succeed in this objective is to gather detailed information of the chemical composition of the side streams. In a previous paper we have described the proximate composition, mineral content, and fatty acid profile of bioethanol post-fermentation corn oil and thin stillage [11].

In this study we report the results of a characterization study focused on the unsaponifiable lipid fraction of the two side-streams. Plant sterols, tocopherols, tocotrienols, squalene, and carotenoids have been analyzed in different lots of post-fermentation corn oil and thin stillage, collected over a 1-year period at a dry-grind corn bioethanol plant.

## 2. Materials and Methods

### 2.1. Collection of Side Streams

Post-fermentation corn oil and thin stillage were obtained from the industrial dry-grind corn bioethanol plant ENVIRAL a.s. (Leopoldov, Slovack Republic). The original feedstock was a yellow non-genetically modified corn (*Zea mays*) grown in the Central East Europe region. Post-fermentation corn oil and thin stillage were sampled from the dry-grind corn ethanol facility as described previously [11].

In the period June 2018–September 2019, approximately at monthly intervals, 1 L corn oil and 3 L thin stillage were delivered (24–48 h from collection) to CREA-Research Centre for Food and Nutrition (Rome, Italy) for analyses. The first samples (lot 1) were obtained from the 2017 corn harvest. The new harvest season started in September 2018 and by October 2018 (lot 2) the newly harvested corn was utilized for the bioethanol production process. Up to eleven lots of corn oil and seven lots of thin stillage have been analyzed. The sampling date at ENVIRAL’s plants of the different side stream lots analyzed in the study are the following: Lot 1 = 29 June 2018, Lot 2 = 22 October 2018, Lot 3 = 26 November 2018, Lot 4 = 9 December 2018, Lot 5 = 18 January 2019, Lot 6 = 25 February 2019, Lot 7 = 22 April 2019, Lot 8 = 23 May 2019, Lot 9 = 21 June 2019, Lot 10 = 29 July 2019, Lot 11 = 26 September 2019.

### 2.2. Chemicals

Pure standards of tocopherols, ergosterol, stigmasterol, campesterol, β-sitosterol, squalene, β–carotene, lutein, zeaxanthin, and β–cryptoxanthin were purchased from Sigma-Aldrich Inc. (St. Louis, MO, USA). Brassicasterol and sitostanol were from AVANTI Polar Lipids Inc. (Alabaster, AL, USA). Tocotrienols were purchased from Cayman Chemical Company (Ann Arbor, MI, USA). All solvents were of analytical or high performance liquid chromatography (HPLC) grade as required. Potassium hydroxide, and butylated hydroxytoluene (BHT) were from Carlo Erba. Tert-butyl-hydroquinone (TBHQ) was from Fluka Chemie AG (Buchs, Switzerland). Deionized water was provided by an Arium^®^ pro UV Water Purification System (Sartorius Stedim Biotech GmbH, Goettingen, Germany).

### 2.3. Sample Treatment

Upon arrival at CREA laboratories, corn oil and thin stillage samples were immediately refrigerated (+4 °C). Corn oil was preserved from light and heat and analyzed without any pre-treatment. Before sampling for analyses, the oil was brought to room temperature and gently shaken in order to re-suspend any solid material sedimented at the bottom of the bottle. Thin stillage was subject to lyophilization before analyses. Total lipids were extracted from the freeze-dried thin stillage (about 5 g) with methanol, chloroform, and water according to the method of Bligh and Dyer [12]. Lipid extracts were analyzed for tocopherols, tocotrienols, plant sterols, and squalene. A specific protocol for extraction of carotenoids from thin stillage was applied as follows. Freeze-dried thin stillage (about 500 mg) was extracted with acetone: methanol (70:30 *v*/*v* containing BHT (500 mg L^−1^) in screw-capped test tubes and allowed to stand 30 min at +3 °C. The mixture was vortexed for 10 s at a high speed (Reax 2000 Vortex mixer, Heidolph, Schwabach, Germany) and centrifuged at 1400× *g*, 5 °C for 15 min and the supernatant was collected. The procedure was repeated until the supernatant and residue were colorless. Extracts were combined, evaporated under vacuum (R-210 Rotavapor™, Bűchi, Switzerland) at 30 °C and brought to a known volume of acetone: methanol (70:30 *v*/*v*).

### 2.4. Chemical Analyses

#### 2.4.1. Analytical Procedures

Analytes in corn oil and extracts of thin stillage were separated and quantified by HPLC before and after saponification to account for the presence of free compounds and of the total amounts released after ester hydrolysis. For analyses of free compounds, direct analyses of diluted corn oil and lipid extracts of thin stillage were accomplished. An aliquot of corn oil or lipid extract was diluted in a known volume of methyl tert-butyl ether (MTBE)/methanol (1:1, *v*/*v*) to be immediately filtered through 0.2-μm syringe filters (Minisart RC4, Sartorius Stedim Biotech) and injected (20 μL) into the HPLC. The total amount of each component, including also the bound fractions released after ester hydrolysis, was determined after saponification. For tocopherols, tocotrienols, plant sterols, and squalene evaluation, saponification in ethanolic potassium hydroxide (10% *w*/*v*) in the presence of TBHQ dissolved in methanol (1% *w*/*v*) occurred at 70 °C for a total of 15 min. For the determination of carotenoid contents, saponification was conducted overnight at room temperature [13]. Saponification occurred in screw-capped ambered test tubes under a nitrogen atmosphere. Unsaponifiables were recovered with n-hexane/ethyl acetate (9:1 *v*/*v*), evaporated to dryness with the aid of a nitrogen stream and dissolved in a suitable amount of MTBE/methanol (1:1, *v*/*v*), to be filtered through 0.2-μm syringe filters before chromatographic injection. All steps were conducted avoiding any direct exposure to light.

#### 2.4.2. High-Performance-Liquid-Chromatography (HPLC)

Chromatographic analyses were carried out on a 1100 Series Agilent HPLC System (Agilent Technologies, Santa Clara, CA, USA) equipped with a quaternary pump, solvent degasser, column thermostat, and photodiode-array (DAD) detector. Tocopherols, tocotrienols, plant sterols, and squalene were determined simultaneously on a reversed-phase Ultrasphere C-18 column (25 cm × 4.6 mm inner diameter, 5 µm, Beckman, Palo Alto, CA., USA) coupled with a C18 guard column (15 cm × 4.6 mm, 5 µm). The mobile phase consisted of acetonitrile/methanol (50:50, *v*/*v*) in isocratic conditions at a flow rate of 1.5 mL min^−1^. Runs were monitored at 215 nm and 282 nm and thermostated at 25 °C. Baseline separation of analytes was accomplished except the pairs β-tocopherol/γ-tocopherol, β-tocotrienol/γ-tocotrienol, campesterol/stigmasterol that coeluted.

Separation of carotenoids occurred on a reversed-phase C-30 column (25 cm × 4.6 mm inner diameter, 5 µm) coupled with a C30 guard cartridge (10 mm, 4 mm, particle size 5 μm), both from YMC Co., Ltd. (Basel, Switzerland). The mobile phase consisted of methanol (eluent A), MTBE (eluent B), and water (eluent C). The gradient program was as follows: time 0: 81% A—15% B—4% C, time 90 min: 7% A—90% B—3% C. Flow rate was 0.7 mL min^−1^ and the column temperature was kept constant at 25 °C. Injection volume was 20 µL. Carotenoids were integrated at 450 nm. Chromatograms were also registered at 325 nm to monitor the elution of steryl ferulate esters in direct extracts. Ultraviolet-visible spectra were recorded over the range 250–680 nm in steps of 2 nm.

Analytes were identified by comparing retention times and UV–Vis absorption spectra to those of authentic standards. Peak areas were used to determine the analyte concentrations in the samples by reference to standard curves obtained by chromatographing pure substances under identical conditions. Data were analyzed with the Agilent ChemStation Software. The analyte contents are expressed as mg per kg of product.

#### 2.4.3. Liquid-Chromatography-Tandem Mass Spectrometry (LC-MS/MS)

Untreated corn oil and thin stillage extracts were also analyzed in the LC-MS/MS system in order to improve the identification of the detected compounds. Analyses were performed on an Agilent 1200 quaternary pump coupled to a 6410 series triple quadrupole. The ion source was an APCI operated in positive mode. Chromatographic separation was conducted on an ACME C18-120A, 100 mm × 2.1 mm column, with 3 µm particle size. Mobile phase was acetonitrile/methanol (50:50, *v*/*v*) in isocratic conditions, at a flow rate of 300 µL min^−1^. The APCI ionization parameters were as follows: Gas temperature 350 °C, vaporizer 375 °C, gas flow 6 L min^−1^, nebulizer 60 psi, capillary voltage 3000 V and corona current 8 µA. Because of a the higher sensibility of the MS/MS detector regarding to the DAD, the concentration of sitostanol in the samples was measured in this system. For doing it, the triple quadrupole analyzer was operated in the MRM mode, with precursor ion 399.7 (corresponding to the molecular weight with the loss of a water molecule and an H^+^ gain) and the following product ions: 95.1, used for quantification, 135.1, 109.1, and 81.1. In all cases the collision energy was 29 V, with the exception of transition 399.1 → 81.1, where it was set at 45 V. Quantification was performed by interpolation on a calibration curve constructed with pure sitostanol standard analyzed in the same conditions, in the range of 0.04 to 1.0 µg mL^−1^.

### 2.5. Quality Assurance

The concentrations of stock solutions of pure standards were determined spectrophotometrically using their specific absorption coefficients. External linear calibration curves of analytical standards, with a minimum of five concentration levels, were constructed for each analyte. The DAD response for each analyte was linear within the calibration ranges with correlation coefficients exceeding 0.998. Repeatability was estimated by calculating the coefficient of variation (CV) after repeated runs of a standard solution containing each compound at the level found in samples. After HPLC runs, the purity of analytes was checked by matching the UV/Vis spectra of each peak with those of the standards. The standard reference material NIST 3278 (Tocopherols in edible oils) was analyzed for validation of the method and quality control of tocopherol data.

### 2.6. Data Treatment

For each parameter, analyses of single lots of post-fermentation corn oil and thin stillage were performed at least in duplicate. Data for single lots, mean, standard deviation, coefficient of variation (CV), and range of values detected during the experimental period were calculated with Microsoft Excel software, 2013 version.

## 3. Results and Discussion

### 3.1. Phytosterols and Squalene

The sterol profile of post-fermentation corn oil sampled monthly from July 2018 to September 2019 and the mean and standard deviation of all values detected are reported in Table 1.

Beta-sitosterol, sitostanol, campesterol + stigmasterol and ergosterol were the sterols identified, based on the comparison with pure standards for retention time and UV spectra characteristics. Peak identity was confirmed in the LC-MS/MS system by comparing the retention time and ionization and fragmentation pattern with those of pure standards. A sterol eluting in correspondence of the retention time of brassicasterol was tentatively identified as δ-5-avenasterol based on LC-MS/MS data, UV spectrum, and literature indications [14,15]. Other minor peaks with UV spectrum and MS/MS ionization/fragmentation characteristics compatible with phytosterols were detected.

Analyses carried out before and after oil saponification allowed the quantification of free and total sterols released after hydrolysis of esters with fatty acids or phenolic acids. The sum of the identified free sterols in direct analysis of corn oil ranged from about 5700 to 8383 mg kg^−1^ in the lots examined. These values more than doubled after saponification (15832–17912 mg kg^−1^), indicating that at least 50% of the sterols was in bound form.

Beta-sitosterol was the most abundant sterol in corn oil, with values as high as 6742–7652 mg kg^−1^ after saponification (corresponding to 42.3–47.4% of total sterols). Sitostanol, the saturated equivalent of sitosterol, was detected at very low amounts in free form (529–739 mg kg^−1^) while after saponification resulted to be the second most abundant sterol (3134–4829 mg kg^−1^, correspondent to 19.8–27.8% of total sterols). This is an indication that sitostanol is mostly present in post-fermentation corn oil in esterified form, in line with the literature on the sterol profile of corn, indicating stanols as highly present in endosperm and bran mainly as ferulate esters [14,16,17,18]. Campesterol + stigmasterol (2041–2616 mg kg^−1^, 13.4–15.4% of total sterols) and the sterol tentatively identified as δ-5-avenasterol (2066–2796 mg kg^−1^, 12.9–15.7% of total sterols) were present in significant amounts in the saponified extract, about half of which in free form. Ergosterol, not inherently present in corn, but essential component of yeast cells membrane, was presumably found in corn oil as a result of the fermentation process. Its levels, ranging from 259 to 461 mg kg^−1^ (corresponding to 1.7–2.4% of total sterols) were not affected by saponification, meaning that this sterol is present mostly in free form. Squalene was present in corn oil at concentrations corresponding to 745–940 mg kg^−1^.

Results obtained on post-fermentation corn oil show that plant sterols are present at considerable levels in this side stream. Compared to a commercial corn oil, one of the richest sources of phytosterols among vegetable oils, with as high as 0.7–0.8% *w*/*w* content of phytosterols [19,20,21,22], post-fermentation corn oil showed much higher sterol levels. This is due to the fact that while a commercial corn oil originates from the germ fraction, post-fermentation corn oil derives from the whole kernel and therefore retains the whole set of phytosterols, phytostanols, and their ferulate esters highly present in the aleurone, pericarp, and endosperm fractions. Moreover, the ethanol produced during fermentation acts as an extractant of sterols and other fat-soluble compounds from the whole fermenting mass, including yeast cells, as evident from the presence of ergosterol, the prevalent sterol in the cell membranes of yeasts, virtually absent in corn. Phytosterol and squalene contents in the different lots examined showed a low variability (CV < 15%), indicating a stable quality of the feedstock and standardized process conditions in the bioethanol plant over the period of study.

Thin stillage is a liquid stream generated in large amounts by the corn dry grind ethanol industry after centrifugation of heavy stillage. Although the majority of undissolved solids are removed with centrifugation, thin stillage still contains, along with a large part of water (90–93%), a residual lipid fraction quantified in the range 1.5–2.3% [11]. The HPLC profile of the unsaponifiable lipid fraction components of thin stillage extracts has shown a qualitative profile comparable to that of corn oil. Chromatographic analyses were performed after saponification. The sterol profile of thin stillage extracts is reported in Table 2, where the values are reported both on a wet mass and on a dry mass basis.

As observed for corn oil, thin stillage contained β-sitosterol as the prevalent sterol (88.3–170 mg kg^−1^ on a wet mass basis), followed by sitostanol (47.4–86.6 mg kg^−1^), campesterol + stigmasterol (29.5–54.0 mg kg^−1^), a sterol tentatively identified as δ-5-avenasterol (18.0–61.3 mg kg^−1^) and trace amounts of ergosterol (4.87–11.3 mg kg^−1^). The percent distribution of single sterols in post-fermentation corn oil and thin stillage were similar as can be seen in Figure S1. In absolute terms, the sterol content of thin tillage is very low on a wet mass basis (205–322 mg kg^−1^ wet mass) compared to corn oil (15832–17912 mg kg^−1^). Squalene, at levels comprised between 10.1 and 19.2 mg kg^−1^ wet mass, was also detected in thin stillage.

The perspective to recover plant sterols and squalene from corn bioethanol co-products for further application in food and nutraceutical products adds value and sustainability to the whole fuel ethanol process. Widely known as cholesterol-lowering compounds, plant sterols are currently approved by regulatory agencies (FDA, EFSA) as food ingredients. Plant sterols, stanols, and their esters are nutritionally relevant nutrients because of their abilities to reduce blood cholesterol levels via partial inhibition of intestinal cholesterol absorption, to inhibit the growth of cancer cells, enhance the immune response, and act as anti-inflammatory and anti-oxidant factors [23,24,25]. Free sterols are the physiologically active form, known for their cholesterol-lowering properties made possible by inhibition of cholesterol absorption in the small intestine. The stanols and sterols esterified to phenolic acids present in corn are mostly hydrolyzed in the intestine. Steryl ferulates and hydroxycinnamate esters, are chain-breaking antioxidants and have proven cholesterol-lowering properties [26,27,28,29]. The development of functional food products enriched with plant sterols is a feasible way to provide consumers with novel healthy food products able to lower serum cholesterol levels [30,31]

Squalene, a polyunsaturated triterpene containing six isoprene units, is naturally present in animal and plant organisms, and in yeast, as an intermediate metabolite in the synthesis of sterols. As a minor constituent of food typical of the Mediterranean diet, squalene has been indicated as a key component in the prevention of cardiovascular heart disease, protection from cancer, and aging. Because of its unique properties, (i.e., drug carrier, adjuvant for vaccines, protective against cancer and other disease, skin repairing properties, UV-protecting properties, antibacterial properties, anti-wrinkle properties) squalene is also indicated in several pharmaceutical and cosmetic applications [32,33]. Although shark liver oil is a major source of squalene in nature, the growing concern for the protection of aquatic animals and the accumulation of persistent chemical pollutants at the high levels of the marine food chain, make plant sources of squalene a sustainable and highly attractive alternative.

### 3.2. Tocopherols and Tocotrienols

The tocol profile of post-fermentation corn oil sampled from July 2018 to September 2019 and the average and standard deviation of all values detected are reported in Table 3. Analyses carried out before and after oil saponification allowed the quantification of free and total tocols released after ester hydrolysis. The coelution of β- and γ-homologues of tocopherols and tocotrienols, common in reversed-phase LC systems, is of no relevance in the case of corn, where β-homologues of tocopherols and tocotrienols are known to be absent or negligible [14,34,35].

Tocols in post-fermentation corn oil were found to be present mostly in their free form, as evidenced by the comparison of levels obtained before and after saponification. Gamma-tocotrienol in direct analysis of corn oil coeluted with one or more unidentified compounds not present in the saponified extract, probably one or more different sterol esters, as evidenced by the analysis of peak spectra characteristics and purity. This coelution did not allow to quantify the amount of free γ-tocotrienol and hence the total amounts of free tocotrienols and free tocols.

Post-fermentation corn oil was characterized by the prevalence of tocopherols (981 ± 42.3 mg kg^−1^, corresponding to 72% of total tocols) over tocotrienols (379 ± 33 mg kg^−1^, corresponding to 28% of total tocols), with γ-tocopherol prevailing over α- and δ- homologues, as typical for corn [31,35]. The levels of γ-tocopherol after saponification accounted for an average value of 742 mg kg^−1^, followed by α-tocopherol (216 mg kg^−1^) and very minor amounts of δ-tocopherol (22.4 mg kg^−1^).

Tocols are inherently present in corn, where they play an antioxidant role, protecting the unsaturated fatty acids from oxidation. In particular, tocopherols are concentrated in corn germ, while tocotrienols are preferentially located in the endosperm and in the outer portions of the kernel.

This explains why in post-fermentation corn oil, which derives from the whole kernel, tocopherol levels are comparable to those of an unrefined corn germ oil, while tocotrienol levels are quite higher [21,35].

Values detected in the different lots examined showed quite stable tocol contents and a low variability (CV < 15%), indicating standardized process conditions in the bioethanol plant over the time and a resistance of tocols to the fuel ethanol production conditions. The prevalence of γ-homologues of tocopherols and tocotrienols over α- and δ-homologues here observed is a characteristic feature of corn that may be of interest for final applications [36,37]. In fact, γ-tocopherol is reported to retain higher antioxidant properties compared to α-tocopherol and to act in synergy with it in biological systems, also protecting from inflammation [38].

The data here reported are in accordance with those reported in literature for ethanol-extracted corn kernel oil and co-products of corn bioethanol production [14,39,40].

The tocol profile of thin stillage and the average and standard deviation of values detected in the different lots after saponification of lipid extracts are reported in Table 4. The amounts are expressed both on a wet mass and dry matter basis. Because of its high dilution, in absolute terms thin stillage has a very low concentration of tocols (average value 20.9 mg kg^−1^ thin stillage). As observed for post-fermentation corn oil, the tocol profile of thin stillage was characterized by the presence of tocopherols (73%) dominating over tocotrienols (27%), with γ-homologues prevailing over α- and δ- homologues a. The relative proportions of each tocopherol and tocotrienol homologue identified in post-fermentation corn oil and thin stillage were quite similar, as can be seen in Figure S2. As observed for phytosterols, in absolute terms, the tocol amounts in thin tillage on a wet mass basis were very low compared to post-fermentation corn oil.

Besides retaining vitamin E activity and playing as potent antioxidants, tocols cover multiple functions in biological systems such as gene expression regulation, signal transduction, and modulation of cell functions through modulation of protein–membrane interactions [41]. All tocopherols possess a high antioxidant activity and are important tools in the prevention of cardiovascular disease and cancer. While most of the studies on vitamin E have been first focused on α-tocopherol, the primary form in most living organisms, further evidences have shown that its homologues have superior biological properties that may be useful for prevention and therapy against chronic diseases [42]. Most recently, tocotrienols have raised increasing interest because of their hypocholesterolemic, neuroprotective, anti-thrombotic, and anti-tumor effects, suggesting that they may serve as effective agents in the prevention and/or treatment of cancer and cardiovascular and neurodegenerative diseases [43,44,45,46]. The enrichment of food products with natural extracts rich of tocols and other natural antioxidants and bioactives is the best strategy to ensure that daily requirements are met and at the same time to improve the healthiness and oxidative stability of processed food [47,48].

### 3.3. Carotenoids

Levels of single carotenoids and average and standard deviation of total amounts observed in post-fermentation corn oil are reported in Table 5. The carotenoid profile of post-fermentation corn oil was that typical of corn, with lutein and zeaxanthin, accounting altogether for over 60% of the total carotenoids, as the prevalent molecules. Minor amounts of β-cryptoxanthin and traces of β-carotene were also present. Cis-isomers of lutein and zeaxanthin, most probably resulting from the high temperatures reached during the biotech process, were also observed and quantified as lutein-equivalents. One of these compounds (N.I.C. 6) was the third most concentrated carotenoid, followed by β-cryptoxanthin.

Values detected in the different lots examined showed higher variability than that found for other molecules, which could be due to differences in the feedstock more than to process conditions in the bioethanol plant.

The average amounts of single carotenoids identified in the different lots of thin stillage analyzed and the average and standard deviation of all values, expressed both on a wet mass and on a dry mass basis, are reported in Table 6. The carotenoid profile of thin stillage was qualitatively very similar to that of post-fermentation corn oil while the concentration, as expected, was much lower. As for corn oil, the amount of carotenoids in the different lots of thin stillage analyzed showed high variability.

In cereals, carotenoids are important phytonutrients responsible for the yellow color of the endosperm, where they occur either in free or esterified forms, mostly with palmitic and linoleic acid. In corn the major carotenoids are the xanthophylls zeaxanthin and lutein, isomers differing by the position of a double bond in the β-ionone ring, with minor amounts of β-cryptoxanthin and β-carotene.

In terms of health benefits, carotenoids are powerful antioxidants protecting cells against reactive oxygen species and free radicals, and playing an important role in the maintenance of good health and disease prevention. Several studies have indicated their protective effect against chronic degenerative, inflammatory, metabolic, and age-related diseases and their immunomodulatory properties [49,50,51,52]. In particular lutein and zeaxanthin, the prevalent xanthophylls in corn, are interesting molecules for the food, pharmaceutical, and nutraceutical sectors because of their strong antioxidant properties and their important role in the maintenance of the normal visual function in humans. As essential components of the eye macula, they protect the retina from the oxidative damages responsible of age-related macular degeneration and cataract [53]. Although fresh vegetables and fruits are a rich source of carotenoids in our diet, the formulation of functional food enriched with carotenoids is a suitable and successful strategy to compensate for nutritional losses occurring during the technological processes or to integrate them in food matrixes not inherently rich of these molecules [54,55]

## 4. Conclusions

This study highlights the potentialities of dry-grind corn bioethanol side streams as sustainable sources of bioactive compounds for high-value applications. Chromatographic analyses on post-fermentation corn oil indicated that this fuel ethanol co-product is particularly rich of plant sterols and stanols and also retains the whole set of tocopherols, tocotrienols, and carotenoids originating from the corn kernel.

The huge volumes of thin stillage produced during the dry-grind corn bioethanol process make the perspective to recover valuable molecules therein a very attractive one. Yet, it still represents a challenge as the high dilution of this stream strongly reduces the affordability and sustainability of any recovery and separation process.

The low variability observed during the year of the chemical profile of the two side streams, in spite of the different origin and seasonality of corn feedstock lots and of the complex biotechnological processes, represents an important element for their industrial utilization.

With a circular economy approach the bioactive molecules present in corn bioethanol co-products, valuable for their antioxidant, anti-inflammatory, hypocholesterolemic, anti-aging, and several other beneficial properties, could be recovered through appropriate technologies and re-introduced in productive processes as ingredients of a wide range of high-value functional products in the food, nutraceutical, and cosmeticeutical sectors.

Even though post-fermentation corn oil is currently utilized by biofuel biorefineries as a feedstock for biodiesel production, its full potentials are actually unexploited since molecules other than fatty acids remain unused. A preliminary separation and fractionation of sterols and other unsaponifiables from corn bioethanol oil would therefore not only maximize the efficiency of biodiesel production, actually hindered by the presence of these molecules, but would also add value to the whole biotech process, opening perspectives for the creation of integrated biorefineries and new value chains.

## Figures and Tables

**Table 1:** Free and total amounts of phytosterols and squalene in post-fermentation corn oil from a dry-grind corn ethanol plant. Data refer to individual lots collected at monthly intervals from July 2018 to September 2019 and overall mean, standard deviation (*sd*), and range of values observed (*n* = 11) *.

	Post-Fermentation Corn Oil													
Lot	ERG		AVN ^a^		STG + CAMP		β-SITO		STN		Σ STEROLS		SQUA	
	mg kg^−1^ Corn Oil													
	Free	Total	Free	Total	Free	Total	Free	Total	Free	Total	Free	Total	Free	Total
1	456	461	2081	2796	1178	2193	3073	7253	598	-	7387	-	867	832
2	413	433	1578	2636	1171	2616	3277	7652	529	4574	6968	17,911	875	843
3	408	411	1662	2248	1052	2186	3100	6742	540	-	6762	-	874	837
4	412	408	1618	2708	1113	2418	3289	7418	540	4277	6972	17,229	899	817
5	253	259	986	2131	1156	2358	4172	7238	569	-	7135	-	947	940
6	288	282	1412	2358	1149	2444	3528	7137	695	4638	7071	16,860	882	831
7	381	402	1428	2362	1034	2330	4180	7460	677	4829	7700	17,382	744	745
8	275	297	982	2066	983	2278	2834	6973	625	4379	5700	15,993	817	800
9	320	336	1353	2202	1081	2430	2803	7027	739	4472	6296	16,467	788	764
10	340	371	1107	2382	1022	2436	5250	7509	663	3134	8383	15,832	767	764
11	314	322	1151	2343	1017	2041	3449	6931	-	-	-	-	803	776
**Mean**	**351**	**362**	**1396**	**2385**	**1087**	**2339**	**3541**	**7213**	**617**	**4329**	**7037**	**16,811**	**842**	**814**
*sd*	*66.7*	*66.6*	*333*	*236*	*69.6*	*158*	*730*	*280*	*73.8*	*556*	*731*	*759*	*62.4*	*54.2*
min	253	259	982	2066	983	2041	2803	6742	529	3134	5700	15,832	744	745
max	456	461	2081	2796	1178	2616	5250	7652	739	4829	8383	17,912	947	940

* Details on the origin of corn feedstock and on lot timings are provided in Materials and Methods. Data for each lot represent mean of duplicate measurements. ^a^ tentative identification (AVN), quantified as brassicasterol-equivalent; - not available. ERG ergosterol, AVN Δ5-avenasterol, STG + CAMP stigmasterol + campesterol, β-SITO β-sitosterol, STN sitostanol, SQUA squalene.

**Table 2:** Total amounts of phytosterols and squalene in thin stillage from a dry-grind corn ethanol plant. Data refer to individual lots collected at monthly intervals from July 2018 to April 2019 and overall mean, standard deviation (*sd*), and range of values observed (*n* = 7) *.

	Thin Stillage						
Lot	ERG	AVN ^a^	STG + CAMP	β-SITO	STN	Σ STEROLS	SQUA
	mg kg^−1^ Thin Stillage (Wet Mass Basis)						
1	11.3	61.3	54.0	170	-	-	19.2
2	5.16	31.4	29.5	88.3	50.6	205	10.1
3	8.33	38.0	40.4	135	-	-	16.2
4	7.62	45.6	41.3	141	86.6	322	15.3
5	5.20	35.8	34.8	103	-	-	12.9
6	5.12	32.2	30.1	110	63.1	241	12.0
7	4.87	18.0	35.0	101	47.4	207	11.4
**Mean**	**6.80**	**37.5**	**37.9**	**121**	**61.9**	**244**	**13.9**
*sd*	*2.41*	*13.4*	*8.43*	*28.6*	*17.8*	*54.8*	*3.17*
min	4.87	18.0	29.5	88.3	47.4	205	10.1
max	11.3	61.3	54.0	170	86.6	322	19.2
	**mg kg^−1^ Thin Stillage (Dry Mass Basis)**						
1	141	767	676	2134	-	-	240
2	63.7	388	364	1090	625	2532	125
3	106	483	514	1718	-	-	206
4	88.2	528	478	1632	1002	3728	178
5	60.9	420	409	1210	-	-	151
6	62.5	394	368	1349	771	2944	146
7	54.6	202	393	1138	532	2320	128
**Mean**	**82.4**	**455**	**457**	**1467**	**732**	**2881**	**168**
*sd*	*31.7*	*172*	*112*	*379*	*205*	*621*	*42.7*
min	54.6	202	364	1090	532	2320	125
max	141	767	676	2134	1002	3728	240

* Details on the origin of corn feedstock and on lot timings are provided in Materials and Methods. Data for each lot represent the mean of duplicate measurements. ^a^ tentative identification (AVN), quantified as brassicasterol-equivalent; - not available. ERG ergosterol, AVN Δ5-avenasterol, STG + CAMP stigmasterol + campesterol, β-SITO β-sitosterol, STN sitostanol, SQUA squalene.

**Table 3:** Free and total amounts of tocopherols (T) and tocotrienols (T3) in post-fermentation corn oil from a dry-grind corn ethanol plant. Data refer to individual lots collected at monthly intervals from July 2018 to September 2019 and overall mean, standard deviation (*sd*), and range of values observed (*n* = 11) *.

			Post-Fermentation Corn Oil															
Lot	α-T		γ-T ^a^		δ-T		Σ T		α-T3		γ-T3 ^a^		δ-T3		Σ T3		Σ (T + T3)	
	mg kg^−1^ Corn Oil																	
	Free	Total	Free	Total	Free	Total	Free	Total	Free	Total	Free	Total	Free	Total	Free	Total	Free	Total
1	179	175	700	706	25.2	18.2	904	899	216	133	---	175	0.00	8.13	-	316	-	1215
2	195	204	753	773	24.8	25.6	973	1004	220	154	---	196	0.00	8.45	-	358	-	1362
3	224	222	748	733	22.4	17.2	994	972	259	185	---	214	0.00	6.08	-	405	-	1377
4	210	216	789	775	24.0	24.4	1023	1016	226	189	---	241	0.00	7.72	-	438	-	1454
5	205	206	733	711	23.5	20.9	962	938	220	183	---	214	0.00	8.31	-	406	-	1344
6	224	212	737	701	21.6	26.1	982	939	220	178	---	202	0.00	4.10	-	384	-	1323
7	224	238	725	766	23.3	21.6	972	1026	208	174	---	203	0.00	9.56	-	386	-	1412
8	201	224	727	731	28.3	30.1	956	985	196	175	---	210	0.00	8.52	-	394	-	1379
9	188	235	694	716	22.3	17.1	904	969	181	168	---	189	0.00	8.80	-	365	-	1334
10	219	224	761	761	21.1	22.0	1002	1006	227	156	---	193	0.00	5.23	-	354	-	1360
11	194	220	761	793	20.6	22.9	975	1036	169	162	---	186	0.00	9.63	-	357	-	1393
**Mean**	**206**	**216**	**739**	**742**	**23.4**	**22.4**	**968**	**981**	**213**	**169**	**---**	**202**	**0.00**	**7.68**	**-**	**379**	**-**	**1359**
*sd*	*15.7*	*17.0*	*27.8*	*32.3*	*2.18*	*4.04*	*36.6*	*42.3*	*24.3*	*16.6*	*---*	*17.9*	*0.00*	*1.78*	*-*	*33.0*	*-*	*60.5*
min	179	175	694	701	20.6	17.1	904	899	169	133	---	175	0.00	4.10	-	316	-	1215
max	224	238	789	793	28.3	30.1	1023	1036	259	189	---	241	0.00	9.63	-	438	-	1454

* Details on the origin of corn feedstock and on lot timings are provided in Materials and Methods. Data for each lot represent the mean of duplicate measurements. ^a^ may contain low or trace amounts of β-homologue; --- data not available for the presence of co-eluting compound(s); - not calculated because free γ-T3 is missing.

**Table 4:** Total amounts of tocopherols (T) and tocotrienols (T3) in thin stillage from a dry-grind corn ethanol plant. Data refer to individual lots collected at monthly intervals from July 2018 to April 2019 and overall mean, standard deviation (*sd*), and range of values observed (*n* = 7) *.

	Thin Stillage								
Lot	α-T	γ-T ^a^	δ-T	Σ T	α-T3	γ-T3 ^a^	δ-T3	Σ T3	Σ T + T3
	mg kg^−1^ Thin Stillage Wet Mass								
1	3.95	16.5	0.57	21.0	2.99	3.96	0.05	6.99	28.0
2	1.89	8.21	0.22	10.3	1.32	2.00	0.00	3.32	13.6
3	3.63	14.0	0.29	17.9	3.14	4.12	0.00	7.26	25.2
4	3.77	13.6	0.39	17.8	3.11	3.90	0.00	7.02	24.8
5	2.70	9.80	0.40	12.9	2.30	2.90	0.00	5.20	18.1
6	2.87	10.3	0.26	13.4	2.31	2.83	0.00	5.14	18.5
7	2.85	10.5	0.46	13.8	2.00	2.55	0.00	4.55	18.4
**Mean**	**3.09**	**11.8**	**0.37**	**15.3**	**2.45**	**3.18**	**0.01**	**5.64**	**20.9**
*sd*	*0.73*	*2.92*	*0.12*	*3.70*	*0.68*	*0.82*	*0.02*	*1.49*	*5.11*
min	1.89	8.21	0.22	10.3	1.32	2.00	0.00	3.32	13.6
max	3.95	16.5	0.57	21.0	3.14	4.12	0.05	7.26	28.0
	**mg kg^−1^ Thin Stillage Dry Mass**								
1	49.4	206	7.15	263	37.4	49.6	0.61	87.5	350
2	23.3	101	2.73	127	16.3	24.7	0.00	41.0	168
3	46.2	178	3.72	228	40.0	52.4	0.00	92.4	320
4	43.6	158	4.51	206	36.0	45.2	0.00	81.2	287
5	31.5	116	4.80	152	26.7	34.2	0.00	60.9	213
6	35.1	125	3.12	163	28.2	34.6	0.00	62.8	226
7	31.9	118	5.16	155	22.4	28.6	0.00	51.0	206
**Mean**	**37.3**	**143**	**4.46**	**185**	**29.6**	**38.5**	**0.09**	**68.1**	**253**
*sd*	*9.39*	*38.5*	*1.48*	*48.6*	*8.65*	*10.7*	*0.23*	*19.4*	*67.0*
min	23.3	101	2.73	127	16.3	24.7	0.00	41.0	168
max	49.4	206	7.15	263	40.0	52.4	0.61	92.4	351

* Details on the origin of corn feedstock and on lot timings are provided in Materials and Methods. Data for each lot represent the mean of duplicate measurements. ^a^ may contain low or trace amounts of β-homologue.

**Table 5:** Free and total amounts of carotenoids in post-fermentation corn oil from a dry-grind corn ethanol plant. Data refer to individual lots collected at monthly intervals from July 2018 to April 2019 and overall mean, standard deviation (*sd*), and range of values observed (*n* = 6) *.

Post-Fermentation Corn Oil																				
Lot	Lutein		Zeaxanthin		β-cryptoxanthin		N.I.C. 1		N.I.C. 2		N.I.C. 3		N.I.C. 4		N.I.C. 5		N.I.C. 6		Total	
mg kg^−1^ Corn Oil																				
	Free	Total	Free	Total	Free	Total	Free	Total	Free	Total	Free	Total	Free	Total	Free	Total	Free	Total	Σ Free	Σ Total
1	68.1	84.4	53.7	69.8	14.3	18.0	9.60	12.1	7.17	8.31	10.5	13.2	6.66	8.96	6.40	8.26	19.6	24.2	196	240
2	64.4	87.0	47.2	75.4	11.8	19.6	9.14	13.9	5.73	8.18	10.5	15.2	6.01	9.05	5.87	8.28	14.1	24.4	175	254
3	88.7	93.8	66.9	72.4	16.9	20.4	12.6	13.1	8.79	9.46	13.7	13.7	7.54	8.85	7.08	7.66	21.2	25.4	243	257
4	80.5	79.4	64.3	61.9	18.3	18.9	10.8	13.2	7.58	7.79	12.5	12.3	7.18	7.79	6.64	7.01	21.1	21.6	229	223
6	84.0	84.4	61.7	62.8	17.9	19.8	11.6	11.7	7.82	8.29	11.9	12.0	7.31	8.55	6.90	7.44	19.1	21.7	228	229
7	88.3	-	58.7	-	16.2	-	10.1	-	6.71	-	11.4	-	8.36	-	8.20	-	21.0	-	229	-
**Mean**	**79.0**	**85.8**	**58.7**	**68.5**	**15.9**	**19.3**	**10.6**	**12.8**	**7.3**	**8.41**	**11.8**	**13.3**	**7.17**	**8.64**	**6.85**	**7.73**	**19.3**	**23.4**	**217**	**240**
*sd*	*10.4*	*5.28*	*7.27*	*5.92*	*2.46*	*0.95*	*1.28*	*0.89*	*1.04*	*0.63*	*1.23*	*1.29*	*0.80*	*0.51*	*0.79*	*0.55*	*2.73*	*1.71*	*25.6*	*14.9*
min	64.4	79.4	47.2	61.9	11.8	18.0	9.14	11.7	5.73	7.79	10.5	12.0	6.01	7.79	5.87	7.01	14.0	21.6	175	223
max	88.7	93.8	66.9	75.4	18.3	20.4	12.6	13.9	8.79	9.46	13.7	15.2	8.36	9.05	8.20	8.28	21.2	25.4	243	257

* Details on the origin of corn feedstock and on lot timings are provided in Materials and Methods. Data for each lot represent the mean of triplicate measurements. N.I.C. 1–6, not identified cis-isomers of carotenoids quantified as lutein-equivalent (in order of elution); - not available.

**Table 6:** Free and total amounts of carotenoids in thin stillage from a dry-grind corn ethanol plant. Data refer to individual lots collected at monthly intervals from July 2018 to April 2019 and overall mean, standard deviation (*sd*), and range of values observed (*n* = 7) *.

	Thin Stillage																			
Lot	Lutein		Zeaxanthin		β-cryptoxanthin		N.I.C. 1		N.I.C. 2		N.I.C. 3		N.I.C. 4		N.I.C. 5		N.I.C. 6		Total	
	Free	Total	Free	Total	Free	Total	Free	Total	Free	Total	Free	Total	Free	Total	Free	Total	Free	Total	Σ Free	Σ Total
	mg kg^−1^ Thin Stillage Wet Mass																			
1	2.62	2.98	2.61	3.23	0.54	0.75	0.30	0.40	0.20	0.23	0.36	0.42	0.24	0.27	0.23	0.27	0.73	0.88	7.84	9.44
2	1.68	2.11	1.39	2.24	0.27	0.62	0.19	0.23	0.07	0.17	0.20	0.40	0.15	0.19	0.14	0.18	0.41	0.61	4.50	6.76
3	2.15	2.78	1.91	3.16	0.39	0.83	0.3	0.37	0.1	0.24	0.19	0.44	0.19	0.23	0.17	0.23	0.58	0.87	5.96	9.14
4	2.28	2.76	2.04	3.04	0.41	0.81	0.29	0.33	0.1	0.23	0.18	0.41	0.19	0.23	0.17	0.24	0.58	0.85	6.24	8.91
5	2.19	2.45	2.09	2.42	0.42	0.63	0.24	0.29	0.19	0.21	0.32	0.38	0.17	0.20	0.16	0.20	0.50	0.66	6.28	7.45
6	2.35	2.6	2.15	2.48	0.47	0.66	0.29	0.35	0.21	0.24	0.33	0.38	0.21	0.25	0.2	0.25	0.60	0.75	6.81	7.95
7	2.07	2.31	2.03	2.25	0.46	0.59	0.19	0.25	0.16	0.17	0.27	0.33	0.18	0.21	0.18	0.22	0.54	0.66	6.09	6.98
**Mean**	**2.19**	**2.57**	**2.03**	**2.69**	**0.42**	**0.70**	**0.26**	**0.32**	**0.15**	**0.21**	**0.26**	**0.39**	**0.19**	**0.23**	**0.18**	**0.23**	**0.56**	**0.75**	**6.25**	**8.09**
*sd*	*0.28*	*0.30*	*0.36*	*0.44*	*0.08*	*0.09*	*0.05*	*0.06*	*0.06*	*0.03*	*0.08*	*0.04*	*0.03*	*0.03*	*0.03*	*0.03*	*0.1*	*0.12*	*1.00*	*1.08*
min	1.68	2.11	1.39	2.24	0.27	0.59	0.19	0.23	0.07	0.17	0.18	0.33	0.15	0.19	0.14	0.18	0.41	0.61	4.50	6.76
max	2.62	2.98	2.61	3.23	0.54	0.83	0.30	0.40	0.21	0.24	0.36	0.44	0.24	0.27	0.23	0.27	0.73	0.88	7.84	9.44
	**mg kg^−1^ Thin Stillage Dry Mass**																			
1	32.7	37.4	32.7	40.4	6.81	9.37	3.82	4.99	2.52	2.91	4.53	5.31	2.98	3.44	2.88	3.34	9.11	10.99	98	118
2	20.8	26.1	17.1	27.7	3.34	7.69	2.38	2.84	0.87	2.15	2.46	4.93	1.83	2.30	1.69	2.25	5.00	7.49	56	83
3	27.4	35.4	24.2	40.2	4.95	10.5	3.77	4.70	1.22	3.00	2.36	5.61	2.36	2.95	2.11	2.90	7.40	11.1	76	116
4	26.4	31.9	23.6	35.2	4.72	9.33	3.35	3.88	1.15	2.64	2.04	4.75	2.18	2.71	2.02	2.78	6.75	9.89	72	103
5	25.8	28.7	24.5	28.4	4.94	7.43	2.80	3.45	2.21	2.49	3.77	4.44	2.02	2.37	1.91	2.35	5.82	7.70	74	87
6	28.7	31.8	26.2	30.3	5.68	8.08	3.59	4.22	2.62	2.89	4.05	4.65	2.58	3.03	2.42	3.04	7.28	9.16	83	97
7	23.3	26.0	22.7	25.3	5.20	6.62	2.16	2.78	1.78	1.86	3.06	3.69	2.05	2.39	1.97	2.42	6.07	7.40	68	78
**Media**	**26.4**	**31.0**	**24.5**	**32.5**	**5.09**	**8.43**	**3.12**	**3.83**	**1.77**	**2.56**	**3.18**	**4.77**	**2.29**	**2.74**	**2.14**	**2.73**	**6.77**	**9.10**	**75**	**98**
*sd*	*3.83*	*4.39*	*4.63*	*6.13*	*1.05*	*1.35*	*0.68*	*0.86*	*0.70*	*0.43*	*0.95*	*0.62*	*0.39*	*0.42*	*0.39*	*0.40*	*1.33*	*1.61*	*13*	*16*
min	20.8	26.0	17.1	25.3	3.34	6.62	2.16	2.78	0.87	1.86	2.04	3.69	1.83	2.30	1.69	2.25	5.00	7.40	56	78
max	32.7	37.4	32.7	40.4	6.81	10.5	3.82	4.99	2.62	3.00	4.53	5.61	2.98	3.44	2.88	3.34	9.11	11.1	98.1	118

* Details on the origin of corn feedstock and on lot timings are provided in Materials and Methods. Data for each lot represent the mean of triplicate measurements. N.I.C. 1–6, not identified cis-isomers of carotenoids quantified as lutein-equivalent (in order of elution).

## Supplementary Materials

The following are available online at https://www.mdpi.com/2304-8158/9/12/1788/s1. Figure S1: Relative distribution of phytosterols in post-fermentation corn oil and thin stillage from a dry-grind corn ethanol plant. Figure S2: Relative distribution of tocopherols (T) and tocotrienols (T3) in post-fermentation corn oil and thin stillage from a dry-grind corn ethanol plant.
