# Supplementary material for: Corn Bioethanol Side Streams: A Potential Sustainable Source of Fat-Soluble Bioactive Molecules for High-Value Applications
Source: Foods. 2020 Dec 2;9(12):1788. doi: 10.3390/foods9121788 (PMC7760800; doi:10.3390/foods9121788)
Supplement: Supplementary file 1 [file foods-09-01788-s001.pdf]

Corn Bioethanol Side Streams: A Potential Sustainable Source of Fat-Soluble Bioactive Molecules for High-Value Applications

Gabriella Di Lena*, Jose Sanchez del Pulgar, Ginevra Lombardi Boccia, Irene Casini, Stefano Ferrari Nicoli

CREA Research Centre for Food and Nutrition, Via Ardeatina 546, 00178 Rome, Italy; jose.sanchezdelpulgar@crea.gov.it (J.S.d.P); g.lombardiboccia@crea.gov.it (G.L.B.); irene.casini@crea.gov.it (I.C.); stefano.nicoli@crea.gov.it (S.F.N.)

*Correspondence: gabriella.dilena@crea.gov.it; Tel.: +39-06-51494445

Figure S1. Relative distribution of phytosterols in post-fermentation corn oil and thin stillage from a dry-grind corn ethanol plant. Data refer to mean values obtained after saponification of 11 lots of corn oil and 7 lots of thin stillage collected at monthly intervals from June 2018 to September 2019. Legend: ERG ergosterol, AVN Δ5-avenasterol, STG+CAMP stigmasterol + campesterol, β-SITO β-sitosterol, STN sitostanol.

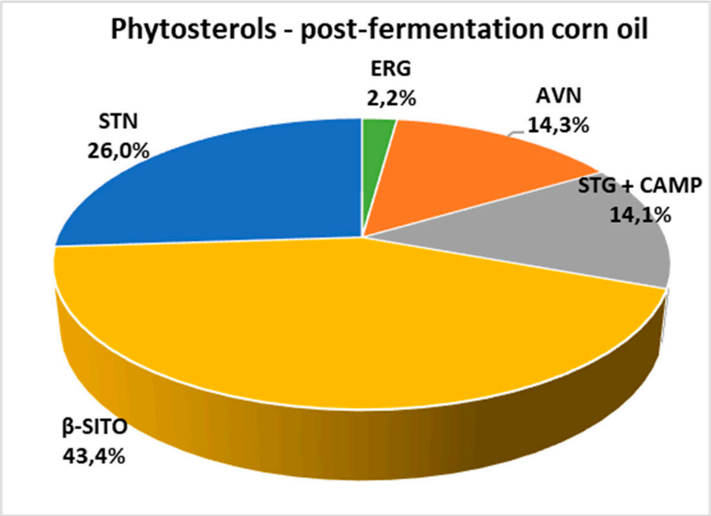

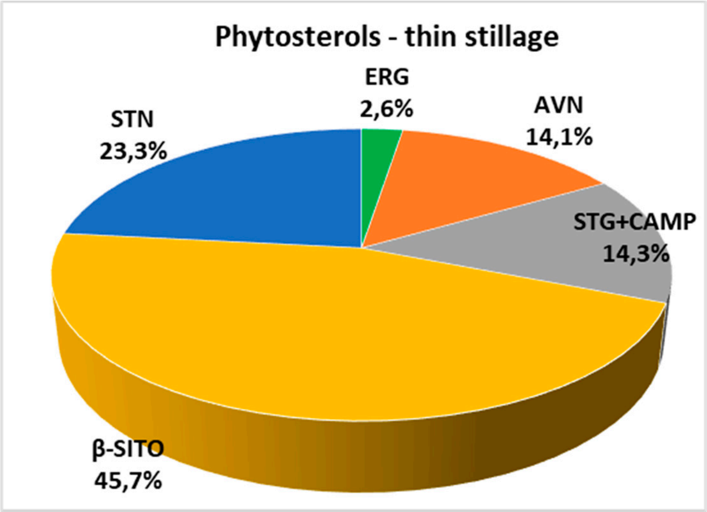

Figure S2. Relative distribution of tocopherols (T) and tocotrienols (T3) in post-fermentation corn oil and thin stillage from a dry-grind corn ethanol plant. Data refer to mean values obtained after saponification of 11 lots of corn oil and 7 lots of thin stillage collected at monthly intervals from June 2018 to September 2019.

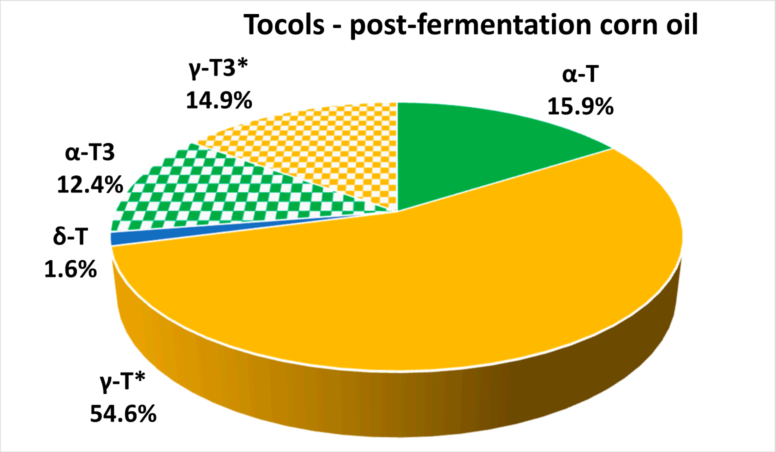

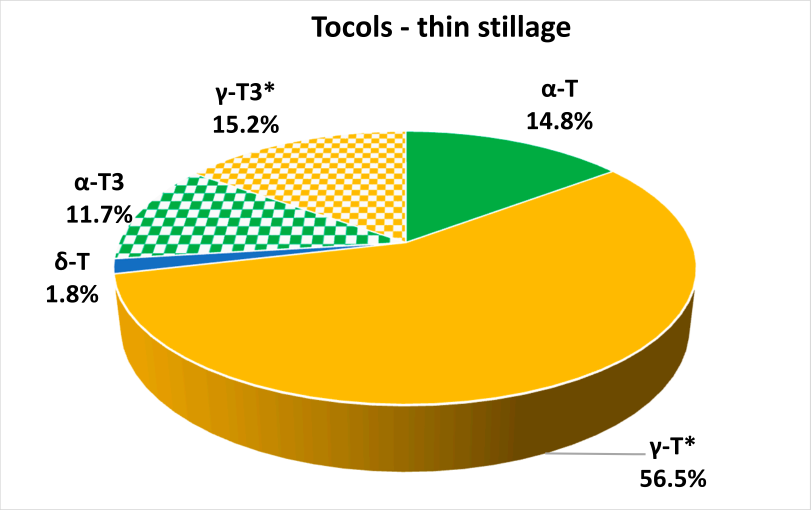

*may contain low or trace amounts of β-homologue
